# NaAg_2_Mo_3_O_9_AsO_4_

**DOI:** 10.1107/S160053681003463X

**Published:** 2010-09-04

**Authors:** Hamadi Hamza, Mohamed Faouzi Zid, Ahmed Driss

**Affiliations:** a Laboratoire de Matériaux et Cristallochimie, Faculté des Sciences de Tunis, Université de Tunis El Manar, 2092 Manar II Tunis, Tunisia

## Abstract

The title compound, sodium disilver arsenatotrimolybdate, Na_0.93 (1)_Ag_2.07 (1)_Mo_3_AsO_13_, was prepared by a solid-state reaction. In the crystal structure, isolated AsO_4_ tetra­hedra share corners with groups of three edge-sharing MoO_6_ octa­hedra. This arrangement leads to the formation of anionic ^1^_∞_[Mo_3_AsO_13_]_*n*_ ribbons extending parallel to [100]. The three metal sites show occupational disorder by Ag^I^ and Na^I^ cations, each with a different Ag:Na ratio. The metal cations are situated in the space between the ribbons and are surrounded by terminal O atoms of the ribbons in the form of distorted *M*O_7_ polyhedra (*M* = Ag, Na) for distances < 3.0 Å. The title compound shows weak ionic conductivity. Structural relationships between different compounds in the quaternary systems *M*–Sb–P–O, *M*–Nb–P–O and *M*–Mo–As–O (*M* is Ag or an alkali metal) are also discussed.

## Related literature

For literature on framework structures containing *M*O_6_ and *X*O_4_ (*M* = transition metal, *X* = P, As) building blocks, see: Benhamada *et al.* (1992 ▶); Harrison *et al.* (1994 ▶); Piffard *et al.* (1985 ▶); Haddad *et al.* (1988 ▶); Ledain *et al.* (1997 ▶). For synthetic details, see: Zid & Jouini (1996 ▶); Zid *et al.* (1998 ▶); Hajji *et al.* (2005 ▶); Hajji & Zid (2006 ▶); Ben Hlila *et al.* (2009 ▶). For structurally related compounds, see: Guyomard *et al.* (1991 ▶); Zid *et al.* (1992 ▶); Lachgar *et al.* (1986 ▶); Ben Amor & Zid (2006 ▶). For details of properties of related compounds, see: Ouerfelli *et al.* (2007 ▶). For the bond-valence model, see: Brown & Altermatt (1985 ▶).

## Experimental

### 

#### Crystal data

NaAg_2_Mo_3_AsO_13_
*M* *_r_* = 814.99
Triclinic, 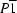

*a* = 8.063 (2) Å
*b* = 8.217 (2) Å
*c* = 9.755 (3) Å
α = 113.42 (2)°
β = 99.49 (1)°
γ = 105.84 (2)°
*V* = 542.6 (3) Å^3^
*Z* = 2
Mo *K*α radiation
μ = 10.17 mm^−1^
*T* = 298 K
0.34 × 0.26 × 0.16 mm

#### Data collection

Enraf–Nonius CAD-4 diffractometer
Absorption correction: ψ scan (North *et al.*, 1968 ▶) *T*_min_ = 0.055, *T*_max_ = 0.194
2895 measured reflections
2371 independent reflections
2282 reflections with *I* > 2σ(*I*)
*R*_int_ = 0.024
2 standard reflections every 120 min  intensity decay: 1.6%

#### Refinement

*R*[*F* ^2^ > 2σ(*F* ^2^)] = 0.030
*wR*(*F* ^2^) = 0.087
*S* = 1.24
2371 reflections
188 parameters
3 restraints
Δρ_max_ = 1.98 e Å^−3^
Δρ_min_ = −1.63 e Å^−3^

### 

Data collection: *CAD-4 EXPRESS* (Duisenberg, 1992 ▶; Macíček & Yordanov, 1992 ▶); cell refinement: *CAD-4 EXPRESS*; data reduction: *XCAD4* (Harms & Wocadlo, 1995 ▶); program(s) used to solve structure: *SHELXS97* (Sheldrick, 2008 ▶); program(s) used to refine structure: *SHELXL97* (Sheldrick, 2008 ▶); molecular graphics: *DIAMOND* (Brandenburg, 1998 ▶); software used to prepare material for publication: *WinGX* (Farrugia, 1999 ▶).

## Supplementary Material

Crystal structure: contains datablocks I, global. DOI: 10.1107/S160053681003463X/wm2387sup1.cif

Structure factors: contains datablocks I. DOI: 10.1107/S160053681003463X/wm2387Isup2.hkl

Additional supplementary materials:  crystallographic information; 3D view; checkCIF report

## Figures and Tables

**Fig. 1.**
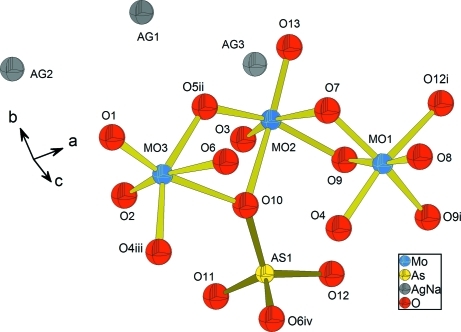
Unité asymétrique dans NaAg2Mo3AsO13. Les ellipsoïdes ont été définis avec 50% de probabilité. [Codes de symétrie: (i) -x, -y + 1, -z + 1; (ii) -x + 1, -y + 1, -z + 2; (iii) x, y, z + 1; (iv)x, y, z - 1.]

**Fig. 2.**
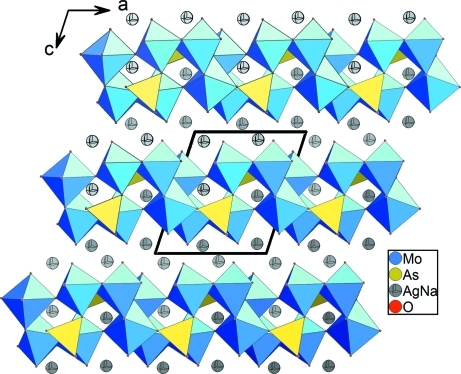
Projection de la stucture de NaAg2Mo3AsO13 selon b, mettant en évidence la disposition des rubans.

**Fig. 3.**
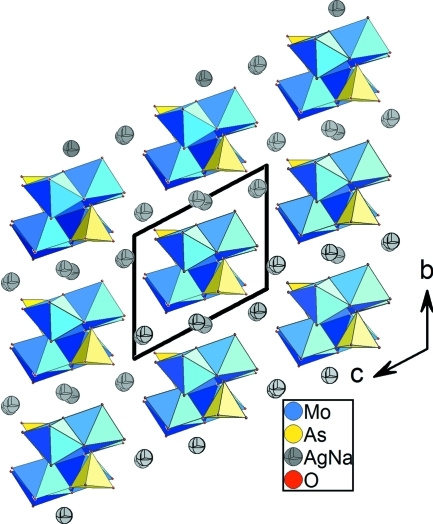
Projection de la stucture de NaAg2Mo3AsO13 selon a, montrant les espaces inter-rubans où résident les cations.

**Fig. 4.**
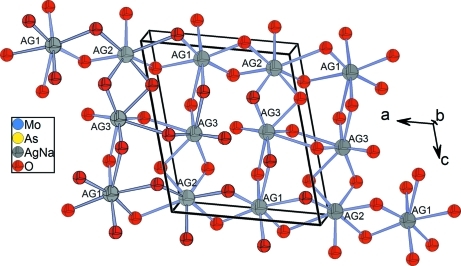
Vue en perspective, selon b, montrant la connection des polyèdres (Ag/Na)O7 dans la stucture de NaAg2Mo3AsO13.

## supplementary crystallographic information

### Comment

La recherche de nouveaux matériaux pouvant être potentiellement des conducteurs ioniques ou bien des échangeurs d'ions, a conduit à s'intéresser aux composés à charpentes mixtes formées d'octaèdres *M*O_6_ (*M* = m\'*et al.* de transition) et de tétraèdres *X*O_4_ (*X* = P, As).

En effet, la jonction entre ces polyèdres conduit à des composés à charpentes ouvertes mixtes présentant de nombreuses propriétés physico-chimiques intéressantes qui sont en relation directe avec leurs structures cristallines (Benhamada *et al.*, 1992; Harrison *et al.*, 1994; Zid *et al.*, 1992; Piffard *et al.*, 1985; Haddad *et al.*, 1988; Ledain *et al.*, 1997). Ce domaine est loin d'être entièrement exploré et fait l'objet des travaux présentant des intérêts fondamentaux que pratiques.

C'est dans ce cadre que nous avons exploré les systèmes *A*–Mo–As–O (*A* = cation monovalent) dans lesquels nous avons précédemment caractérisé plusieurs phases intéressantes: K_2_MoO_2_As_2_O_7_ (Zid & Jouini, 1996), Rb_2_MoO_2_As_2_O_7_ (Zid *et al.*, 1998), Li(MoO_2_)_2_O(AsO_4_) (Hajji *et al.*, 2005) et AgMoO_2_AsO_4_ (Hajji & Zid, 2006). Un nouveau produit de symétrie triclinique a été synthétisé par réaction à l'état solide. Le mode de préparation, la détermination de la structure par diffraction des rayons-X sur monocristal et certaines propriétés physiques seront présentés dans ce travail. La nouvelle phase NaAg_2_Mo_3_AsO_13_ est l'une des rares molybdyloxydes double d'arséniate et de métaux monovalents à charpente unidimensionnelle synthétisée dans ces dernières années.

L'unité anionique asymétrique [Mo_3_AsO_13_]^3-^ dans NaAg_2_Mo_3_AsO_13_ est formée par trois octaèdres MoO_6_, mettant en commun des arêtes, reliés par partage d'un sommet à un tétraèdre AsO_4_ (Fig. 1). Dans la charpente anionique unidimensionnelle, chaque unité se lie à son centrosymétrique, d'une part par mise en commun de sommets entre les octaèdres terminaux des deux unités pour former des cycles à six octaèdres et d'autre part par formation de ponts mixtes As—O—Mo entre octaèdres et tétraèdres appartenant à deux unités anioniques symétriques [Mo_3_AsO_13_]^3-^ différentes. L'association de ces doubles unités par partage d'arêtes conduit à des rubans infinis disposés selon la direction *a* (Fig. 2). Les atomes d'oxygène non engagés dans les ponts pointent vers l'espace inter-rubans où résident les cations Na^+^ et Ag^+^ (Fig. 3). Les moyennes des distances Mo—O, As—O, Ag—O et Na—O dans la structure sont conformes à celles rencontrées dans la littérature (Hajji & Zid, 2006; Ben Hlila *et al.*, 2009). Par contre, si on se limite à une sphère de coordination de rayon égal à 3 Å moyennant le programme *DIAMOND* 2.0 (Brandenburg, 1998), on montre que les polyèdres irréguliers *M*O_7_ (*M* = Ag/Na) se lient par partage d'arêtes pour développer des couches disposées selon les plans (010) (Fig. 4). De plus, le calcul des différentes valences des liaisons utilisant la formule empirique de Brown (Brown & Altermatt, 1985) vérifie bien les valeurs de charges des ions: Mo1 (5,93), Mo2 (5,84), Mo3 (5,85), As (4,81), Ag1 (0,97), Ag2 (1,03), Ag3 (1,09), Na1 (0,88), Na2 (0,93) et Na3 (1,07), attendues dans la phase étudiée.

La structure étudiée étant originale, à notre connaissance, nous avons d'une part sélectioné de la littérature des structures à charpente unidimensionnelle pour présenter les différences essentielles dans la jonction des polyèdres pour conduire à des rubans de nature différente, et d'autre part essayer de trouver une certaine analogie structurale avec des composés ayant un groupement *X*_3_O_11_ (*X* = Nb, Mo ou Sb). En effet, dans la charpente anionique de Na_3_SbO(PO_4_)_2_ (Guyomard *et al.*, 1991), les rubans sont formés au moyen de chaînes d'octaèdres partageant des sommets et reliés à des tétraèdres PO_4_ par mise en commun de sommets. Dans le niobylphosphate K_3_NbO(PO_4_)_2_ (Zid *et al.*, 1992), ils sont construits au moyen de double chaînes classiques NbPO_8_ mettant en commun des sommtes oxygène entre octaèdres et tétraèdres. Dans la charpente unidimensionnelle de K_2_SbO_2_PO_4_ (Lachgar *et al.*, 1986), chaque ruban est formé au moyen d'une chaîne d'octaèdres, partageant des arêtes, dans laquelle un tétraèdre PO_4_ met en commun deux sommets avec repectivement deux octaèdres juxtaposés. Dans le triniobyloxoarséniate de formulation Ag_3_Nb_3_As_2_O_14_ (Ben Amor & Zid, 2006), la charpente anionique se caractérise par la présence des groupements *X*_3_O_11_, formés par trois octaèdres (*X* = Nb) partageant des arêtes, similaires à ceux rencontrés dans notre trimolybdate (*X* = Mo) mais ils sont disposés, contrairement à notre structure, perpendiculairement les uns aux autres. De plus l'association, dans Ag_3_Nb_3_As_2_O_14_, de ces groupements Nb_3_O_11_ par mise en commum de sommets avec les tètraédes AsO_4_ conduit à une structure tridimensionnelle.

Afin d'utiliser les données structurales trouvées, favorables à une bonne mobilité ionique, et les relier aux propriétés physico-chimiques et en particulier de conduction ioniques (Ouerfelli *et al.*, 2007) des mesures de la résistance en fonction de la température de notre matériau ont été réalisées moyennant un pont d'impédance complexe de type HP4192A sur un échantillon pur compacté, sous forme de pastille (e/s = 0,073 cm^-1^). Les valeurs des conductivités obtenues en montée de températures vérifient bien l'un des modèles d'Arrhenius: Ln(σT) = Lnσ_0_ - (10^4^Ea/kT). En effet, cette étude montre que ce matériau, ayant une énergie d'activation égale à 0,647 eV et des conductivités égales à: 1,27 × 10^-6^ S cm^-1^ à 613 K, 2,11 × 10^-6^ S cm^-1^ à 643 K, 3,29 × 10^-6^ S cm^-1^ à 673 K et 5,39 × 10^-6^ S cm^-1^ à 703 K, est classé comme étant un conducteur ionique moyen. Ce résultat est comparable à celui trouvé pour le composé au thallium, rencontré dans la littérature (Ouerfelli *et al.*, 2007).

### Experimental

Les cristaux relatifs à la phase NaAg_2_Mo_3_AsO_13_ ont été obtenus à partir des réactifs: (NH_4_)_2_Mo_4_O_13_ (Fluka, 69858), NH_4_H_2_AsO_4_ (préparé au laboratoire, ASTM 01-775), AgNO_3_ (Fluka, 69658) et Na_2_CO_3_ (Prolabo, 27778) pris dans les rapports molaires Ag:Na:Mo:As égaux à 2:1:3:1. Le mélange, finement broyé, est préchauffé à l'air jusqu'à 623 K en vue d'éliminer les composés volatils. Il est ensuite porté, par palier de 100 degrés, jusqu'à une température de synthèse proche de la fusion, 823 K. Le mélange est alors abandonné à cette température pendant deux semaines pour favoriser la germination et la croissance des cristaux. Le résidu final a subi en premier un refroidissement lent (5°/jour) jusqu'à 773 K puis un second rapide (50°/h) jusqu'à la température ambiante. Des cristaux jaunâtres, de taille suffisante pour les mesures des intensités, ont été séparés du flux par l'eau chaude. Une analyse qualitative au M.E.B.E. de type FEI Quanta 200 d'un cristal confirme la présence des différents éléments chimiques attendus: Ag, Mo, As, Na et l'oxygène.

### Refinement

Dans l'affinement final et pour des raisons de neutralité électrique, les taux d'occupation des cations Na^+^ et Ag^+^ ont été menés en utilisant la condition SUMP autorisée par le programme *SHELX* (Sheldrick, 2008). De plus les ellipsoïdes et les positions des ions Na^+^ ont été définis, moyennant respectivement les conditions EADP et EXYZ autorisée par le programme *SHELX*, identiques à ceux des ions Ag^+^. Les densités d'électrons max et min restants dans la Fourier-différence sont situées respectivements à 0.75 Å de Mo1 et à 0.94 Å de Mo2. Il en résulte la composition chimique finale Ag_2,07 (1)_Na_0,93 (1)_Mo_3_AsO_13_ du nouveau matériau obtenu.

### Crystal data

**Table:**

NaAg_2_Mo_3_AsO_13_	*Z* = 2
*M**_r_* = 814.99	*F*(000) = 741
Triclinic, *P*1	*D*_x_ = 4.989 Mg m^−^^3^
Hall symbol: -P 1	Mo *K*α radiation, λ = 0.71073 Å
*a* = 8.063 (2) Å	Cell parameters from 25 reflections
*b* = 8.217 (2) Å	θ = 10–15°
*c* = 9.755 (3) Å	µ = 10.17 mm^−^^1^
α = 113.42 (2)°	*T* = 298 K
β = 99.49 (1)°	Prism, yellow
γ = 105.84 (2)°	0.34 × 0.26 × 0.16 mm
*V* = 542.6 (3) Å^3^	

### Data collection

**Table:**

Enraf–Nonius CAD-4 diffractometer	2282 reflections with *I* > 2σ(*I*)
Radiation source: fine-focus sealed tube	*R*_int_ = 0.024
graphite	θ_max_ = 27.0°, θ_min_ = 2.4°
ω/2θ scans	*h* = −10→2
Absorption correction: ψ scan (North *et al.*, 1968)	*k* = −10→10
*T*_min_ = 0.055, *T*_max_ = 0.194	*l* = −12→12
2895 measured reflections	2 standard reflections every 120 min
2371 independent reflections	intensity decay: 1.6%

### Refinement

**Table:**

Refinement on *F*^2^	Primary atom site location: structure-invariant direct methods
Least-squares matrix: full	Secondary atom site location: difference Fourier map
*R*[*F*^2^ > 2σ(*F*^2^)] = 0.030	*w* = 1/[σ^2^(*F*_o_^2^) + (0.0402*P*)^2^ + 4.8728*P*] where *P* = (*F*_o_^2^ + 2*F*_c_^2^)/3
*wR*(*F*^2^) = 0.087	(Δ/σ)_max_ = 0.001
*S* = 1.24	Δρ_max_ = 1.98 e Å^−^^3^
2371 reflections	Δρ_min_ = −1.63 e Å^−^^3^
188 parameters	Extinction correction: *SHELXL97* (Sheldrick, 2008), Fc^*^=kFc[1+0.001xFc^2^λ^3^/sin(2θ)]^-1/4^
3 restraints	Extinction coefficient: 0.0306 (10)

### Special details

**Table:**

Geometry. All e.s.d.'s (except the e.s.d. in the dihedral angle between two l.s. planes) are estimated using the full covariance matrix. The cell e.s.d.'s are taken into account individually in the estimation of e.s.d.'s in distances, angles and torsion angles; correlations between e.s.d.'s in cell parameters are only used when they are defined by crystal symmetry. An approximate (isotropic) treatment of cell e.s.d.'s is used for estimating e.s.d.'s involving l.s. planes.
Refinement. Refinement of *F*^2^ against ALL reflections. The weighted *R*-factor *wR* and goodness of fit *S* are based on *F*^2^, conventional *R*-factors *R* are based on *F*, with *F* set to zero for negative *F*^2^. The threshold expression of *F*^2^ > σ(*F*^2^) is used only for calculating *R*-factors(gt) *etc*. and is not relevant to the choice of reflections for refinement. *R*-factors based on *F*^2^ are statistically about twice as large as those based on *F*, and *R*- factors based on ALL data will be even larger.

### Fractional atomic coordinates and isotropic or equivalent isotropic displacement parameters (Å^2^)

**Table:**

	*x*	*y*	*z*	*U*_iso_*/*U*_eq_	Occ. (<1)
Mo1	0.01511 (7)	0.28903 (7)	0.35866 (6)	0.00656 (15)	
Mo2	0.19906 (7)	0.41276 (7)	0.74653 (6)	0.00685 (16)	
Mo3	0.38388 (7)	0.55984 (7)	1.26729 (6)	0.00759 (16)	
As1	0.44918 (8)	0.77640 (8)	0.66832 (7)	0.00632 (17)	
Ag1	0.40570 (9)	0.14320 (9)	0.93581 (8)	0.0194 (3)	0.833 (4)
Na1	0.40570 (9)	0.14320 (9)	0.93581 (8)	0.0194 (3)	0.167 (8)
Ag2	−0.10888 (11)	0.15255 (13)	0.92123 (10)	0.0278 (3)	0.708 (5)
Na2	−0.10888 (11)	0.15255 (13)	0.92123 (10)	0.0278 (3)	0.292 (8)
Ag3	0.24782 (12)	0.97254 (12)	0.47319 (11)	0.0173 (3)	0.527 (4)
Na3	0.24782 (12)	0.97254 (12)	0.47319 (11)	0.0173 (3)	0.473 (8)
O1	0.2795 (7)	0.7125 (7)	1.2563 (6)	0.0184 (10)	
O2	0.2847 (7)	0.3639 (7)	1.0825 (6)	0.0164 (10)	
O3	0.2647 (7)	0.6107 (7)	0.9260 (6)	0.0189 (10)	
O4	0.2389 (6)	0.4348 (6)	0.3654 (5)	0.0115 (9)	
O5	0.3870 (6)	0.3088 (6)	0.7529 (5)	0.0098 (8)	
O6	0.5302 (6)	0.7705 (6)	1.5190 (5)	0.0111 (9)	
O7	0.0860 (6)	0.1790 (7)	0.4672 (6)	0.0142 (9)	
O8	−0.0573 (7)	0.1214 (7)	0.1630 (6)	0.0159 (10)	
O9	0.0571 (6)	0.4905 (6)	0.6238 (5)	0.0113 (9)	
O10	0.4147 (6)	0.5666 (6)	0.6783 (5)	0.0094 (8)	
O11	0.5864 (6)	0.9570 (7)	0.8439 (6)	0.0169 (10)	
O12	0.2510 (6)	0.8156 (6)	0.6336 (6)	0.0117 (9)	
O13	0.0388 (6)	0.2362 (7)	0.7601 (6)	0.0135 (9)	

### Atomic displacement parameters (Å^2^)

**Table:**

	*U*^11^	*U*^22^	*U*^33^	*U*^12^	*U*^13^	*U*^23^
Mo1	0.0066 (3)	0.0069 (3)	0.0065 (3)	0.00209 (19)	0.00062 (19)	0.0042 (2)
Mo2	0.0067 (3)	0.0091 (3)	0.0067 (3)	0.00284 (19)	0.00147 (19)	0.0059 (2)
Mo3	0.0072 (3)	0.0109 (3)	0.0066 (3)	0.0037 (2)	0.00137 (19)	0.0060 (2)
As1	0.0066 (3)	0.0067 (3)	0.0058 (3)	0.0016 (2)	0.0007 (2)	0.0041 (2)
Ag1	0.0228 (4)	0.0176 (4)	0.0158 (4)	0.0095 (3)	0.0055 (3)	0.0050 (3)
Na1	0.0228 (4)	0.0176 (4)	0.0158 (4)	0.0095 (3)	0.0055 (3)	0.0050 (3)
Ag2	0.0221 (5)	0.0427 (6)	0.0234 (5)	0.0058 (4)	0.0113 (3)	0.0224 (4)
Na2	0.0221 (5)	0.0427 (6)	0.0234 (5)	0.0058 (4)	0.0113 (3)	0.0224 (4)
Ag3	0.0183 (5)	0.0123 (5)	0.0218 (5)	0.0039 (3)	0.0041 (3)	0.0106 (4)
Na3	0.0183 (5)	0.0123 (5)	0.0218 (5)	0.0039 (3)	0.0041 (3)	0.0106 (4)
O1	0.016 (2)	0.025 (3)	0.024 (3)	0.012 (2)	0.007 (2)	0.017 (2)
O2	0.019 (2)	0.019 (2)	0.011 (2)	0.0084 (19)	0.0034 (19)	0.0054 (19)
O3	0.028 (3)	0.019 (2)	0.010 (2)	0.009 (2)	0.007 (2)	0.006 (2)
O4	0.0052 (19)	0.016 (2)	0.013 (2)	0.0006 (17)	0.0017 (17)	0.0086 (18)
O5	0.009 (2)	0.015 (2)	0.012 (2)	0.0073 (17)	0.0054 (17)	0.0097 (18)
O6	0.014 (2)	0.013 (2)	0.007 (2)	0.0032 (17)	0.0051 (17)	0.0063 (17)
O7	0.014 (2)	0.020 (2)	0.017 (2)	0.0083 (19)	0.0054 (18)	0.015 (2)
O8	0.018 (2)	0.014 (2)	0.011 (2)	0.0040 (19)	0.0062 (19)	0.0019 (18)
O9	0.009 (2)	0.011 (2)	0.013 (2)	0.0027 (17)	−0.0005 (17)	0.0069 (18)
O10	0.010 (2)	0.009 (2)	0.013 (2)	0.0031 (16)	0.0038 (17)	0.0085 (18)
O11	0.016 (2)	0.015 (2)	0.013 (2)	0.0020 (19)	0.0001 (18)	0.0046 (19)
O12	0.007 (2)	0.013 (2)	0.020 (2)	0.0061 (17)	0.0052 (18)	0.0110 (19)
O13	0.011 (2)	0.019 (2)	0.016 (2)	0.0062 (18)	0.0047 (18)	0.013 (2)

### Geometric parameters (Å, °)

**Table:**

Mo1—O8	1.728 (5)	Mo3—O10^ii^	2.252 (4)
Mo1—O7	1.763 (4)	As1—O11	1.679 (5)
Mo1—O4	1.847 (4)	As1—O6^iv^	1.681 (4)
Mo1—O9^i^	2.003 (4)	As1—O10	1.715 (4)
Mo1—O12^i^	2.109 (4)	As1—O12	1.721 (4)
Mo1—O9	2.356 (5)	Ag1—O2	2.378 (5)
Mo2—O3	1.718 (5)	Ag1—O11^v^	2.414 (5)
Mo2—O13	1.726 (5)	Ag1—O3^ii^	2.586 (5)
Mo2—O9	1.925 (4)	Ag1—O11^ii^	2.586 (5)
Mo2—O5	1.936 (4)	Ag2—O13	2.322 (5)
Mo2—O10	2.217 (4)	Ag2—O11^vi^	2.336 (5)
Mo2—O7	2.452 (5)	Ag2—O8^iii^	2.452 (5)
Mo3—O1	1.717 (5)	Ag3—O6^vii^	2.330 (4)
Mo3—O2	1.731 (5)	Ag3—O12	2.392 (5)
Mo3—O5^ii^	1.954 (4)	Ag3—O7^viii^	2.418 (5)
Mo3—O4^iii^	1.958 (4)	Ag3—O1^iv^	2.447 (5)
Mo3—O6	2.229 (5)	Ag3—O13^i^	2.542 (5)
			
O8—Mo1—O7	106.5 (2)	O5^ii^—Mo3—O4^iii^	153.23 (19)
O8—Mo1—O4	97.9 (2)	O1—Mo3—O6	88.2 (2)
O7—Mo1—O4	99.3 (2)	O2—Mo3—O6	168.1 (2)
O8—Mo1—O9^i^	108.7 (2)	O5^ii^—Mo3—O6	80.51 (18)
O7—Mo1—O9^i^	143.3 (2)	O4^iii^—Mo3—O6	80.69 (18)
O4—Mo1—O9^i^	86.02 (19)	O1—Mo3—O10^ii^	164.6 (2)
O8—Mo1—O12^i^	88.9 (2)	O2—Mo3—O10^ii^	89.2 (2)
O7—Mo1—O12^i^	90.1 (2)	O5^ii^—Mo3—O10^ii^	71.43 (16)
O4—Mo1—O12^i^	166.24 (19)	O4^iii^—Mo3—O10^ii^	86.64 (17)
O9^i^—Mo1—O12^i^	80.47 (17)	O6—Mo3—O10^ii^	79.96 (17)
O8—Mo1—O9	169.15 (19)	O11—As1—O6^iv^	113.4 (2)
O7—Mo1—O9	73.34 (19)	O11—As1—O10	108.0 (2)
O4—Mo1—O9	92.76 (18)	O6^iv^—As1—O10	110.5 (2)
O9^i^—Mo1—O9	70.15 (19)	O11—As1—O12	106.6 (2)
O12^i^—Mo1—O9	80.27 (17)	O6^iv^—As1—O12	106.3 (2)
O3—Mo2—O13	104.5 (2)	O10—As1—O12	112.0 (2)
O3—Mo2—O9	98.4 (2)	O2—Ag1—O11^v^	166.50 (17)
O13—Mo2—O9	103.7 (2)	O2—Ag1—O3^ii^	92.84 (17)
O3—Mo2—O5	106.0 (2)	O11^v^—Ag1—O3^ii^	76.24 (16)
O13—Mo2—O5	96.0 (2)	O2—Ag1—O11^ii^	85.15 (16)
O9—Mo2—O5	143.54 (19)	O11^v^—Ag1—O11^ii^	87.38 (17)
O3—Mo2—O10	90.5 (2)	O3^ii^—Ag1—O11^ii^	92.39 (15)
O13—Mo2—O10	163.3 (2)	O13—Ag2—O11^vi^	125.47 (17)
O9—Mo2—O10	80.67 (18)	O13—Ag2—O8^iii^	142.90 (16)
O5—Mo2—O10	72.53 (17)	O11^vi^—Ag2—O8^iii^	89.43 (17)
O3—Mo2—O7	166.5 (2)	O6^vii^—Ag3—O12	131.29 (16)
O13—Mo2—O7	82.7 (2)	O6^vii^—Ag3—O7^viii^	77.75 (16)
O9—Mo2—O7	68.61 (18)	O12—Ag3—O7^viii^	127.64 (16)
O5—Mo2—O7	84.14 (17)	O6^vii^—Ag3—O1^iv^	98.64 (16)
O10—Mo2—O7	84.01 (16)	O12—Ag3—O1^iv^	92.52 (16)
O1—Mo3—O2	103.3 (2)	O7^viii^—Ag3—O1^iv^	129.80 (17)
O1—Mo3—O5^ii^	97.1 (2)	O6^vii^—Ag3—O13^i^	121.56 (15)
O2—Mo3—O5^ii^	101.0 (2)	O12—Ag3—O13^i^	106.27 (15)
O1—Mo3—O4^iii^	101.2 (2)	O7^viii^—Ag3—O13^i^	74.12 (16)
O2—Mo3—O4^iii^	93.7 (2)	O1^iv^—Ag3—O13^i^	65.54 (16)

Symmetry codes: (i) −*x*, −*y*+1, −*z*+1; (ii) −*x*+1, −*y*+1, −*z*+2; (iii) *x*, *y*, *z*+1; (iv) *x*, *y*, *z*−1; (v) *x*, *y*−1, *z*; (vi) *x*−1, *y*−1, *z*; (vii) −*x*+1, −*y*+2, −*z*+2; (viii) *x*, *y*+1, *z*.
